# Online reviews in primary care – a mimicry indicator of quality? A comparative analysis across urban and rural regions in Germany

**DOI:** 10.1007/s43999-025-00080-2

**Published:** 2025-12-08

**Authors:** Jonas Cittadino, Jost Steinhäuser

**Affiliations:** Institute of Family Medicine, University Medical Center Schleswig-Holstein, Campus Lübeck, Lübeck, Germany

**Keywords:** Primary care, Online reviews, Natural language processing, Patient experience, Regional comparison

## Abstract

**Background:**

Online reviews influence how patients select primary care providers. However, little is known about the language used in such reviews, regional differences in tone or content, and reviewer behavior.

**Objective:**

This study aims to analyze Google reviews of general practices in four selected German regions - two urban (Lübeck, Dresden) and two rural (Main-Tauber District, Mecklenburg Lake Plateau) - to identify linguistic patterns, reviewer characteristics, and implications for quality improvement.

**Methods:**

We extracted all publicly available Google Maps entries for “Hausarzt [City], Deutschland” using an Application Programming Interface (API). Natural Language Processing (NLP) was applied to analyze the content and metadata of reviews. Descriptive statistics and ordinal logistic regression identified word combinations associated with high and low ratings. Results were compared across regions.

**Results:**

A total of 9307 reviews from 1854 practices were analyzed. Ratings were predominantly positive (78.7% 5-star) with no regional differences. Reviewers who wrote positive reviews tended to have written more reviews overall. Common terms in positive reviews included “attentive”, “competent,” and “takes time”; negative reviews featured “unfriendly” and “long wait”. Lexical patterns and predictors were consistent across all regions.

**Conclusion:**

Google reviews provide some insights into patient perceptions of primary care. Key expectations, such as friendliness and attentiveness, appear stable across urban and rural settings, with minimal regional variation in review language.

**Supplementary Information:**

The online version contains supplementary material available at 10.1007/s43999-025-00080-2.

## Introduction

While everyone wants to be treated by a “good” physician, it remains unclear what good means [1]. When given the option, it seems reasonable to seek out information before becoming a patient of a physician you have never met [2, 3]. In recent years Physician Rating Websites (PRW), health-specific portals with structured rating prompts and, in parts, curation, and publicly available platforms like *Google Maps* have become somewhat important in supporting such patients’ healthcare decisions [4–11]. Among these platforms, where health care providers are reviewed alongside other services and prompts are less standardized, *Google Reviews* stands out due to its accessibility, reach, and integration into everyday search behavior.

In Germany, awareness and reading of online health information and rating portals are common, while active contribution of ratings remains comparatively low [5, 12]. Recent surveys, however, report substantial trust and influence on decisions among users of health portals and PRWs [13, 14]. However, little is known about what types of comments are associated with high or low ratings. Even less is known about whether such content varies by region - for example, between urban and rural areas or between former East and West Germany.

To address this gap, this study analyzes *Google reviews* of general practices in four contrasting German regions using Natural Language Processing (NLP). It aims to identify linguistic patterns, reviewer behavior, and regional differences in patient perceptions of primary care.

## Methods

### Region selection

In Germany, the *Bundesinstitut für Bau-*,* Stadt- und Raumforschung* (BBSR) provides a widely used typology for classifying regions as urban or rural based on demographic and spatial indicators [15]. For each former part of Germany (East and West) we selected one urban and one rural region each to reflect different circumstances. We choose the city of Lübeck, the city of Dresden, the rural region of the Main-Tauber District and the rural region of the Mecklenburg Lake Plateau [15].

### Data collection and characteristics

Data were extracted from Google Maps using the Outscraper [16] Online Portal. The portal used the *google_maps_search* endpoint with the query set to “Hausarzt [Region], Deutschland,” using coordinate-based bounding boxes covering each administrative area. For Main-Tauber District and Mecklenburg Lake Plateau, all associated counties were included. The scrape was performed on 30 March 2025 and re-run on 23 October 2025 to validate reproducibility and detect missing/deleted reviews. Practices across runs were harmonized by Google *place_id* normalization. As a query robustness check, we repeated the Lübeck scrape with the alternative term “Hausärztin” under identical parameters.

The extracted data was organized into two structured datasets: (1) Practice Dataset: This dataset contained information at the practice level, including name, number of reviews, average rating, presence of opening hours, and existence of a website. (2) Review Dataset: This dataset contained information at the review level, linking each review to a specific practice. It included the review text, the star rating given, the associated practice ID, and the total number of ratings and reviews submitted by each reviewer across *Google Maps*.

### Natural language processing analysis

All review texts were processed using a Natural Language Processing (NLP) pipeline which included the following steps: (1) Preprocessing: Texts were lowercased, punctuation was removed, and German stopwords were eliminated using the *spaCy* language model [17] (de_core_news_sm) with an additional list of domain-irrelevant terms (e.g. “seit”, “bin”). Lemmatization was applied to reduce words to their base forms [18]. Lemmatization collapses inflected forms to a base form (e.g., *saying/ said* become *say*). Only lemmas with at least three characters were retained. (2) Keyword Extraction: N-grams (unigrams, bigrams and trigrams) were extracted using scikit-learn’s [19] *CountVectorizer*, considering only those appearing in at least ten reviews [20]. (3) Sentiment-Associated Terms: Terms were analyzed separately for reviews with 5-star and 1-star ratings to identify words that frequently co-occurred with either extreme. (4) Feature Preparation: Selected keywords were converted into binary features indicating presence or absence in each review for further regression analysis.

### Rater analysis

The following metadata related to reviewers was analyzed to explore differences between positive and negative raters: activity (total number of reviews submitted by each reviewer across *Google Maps*) and behavioral patterns (assessment of whether reviewers who wrote positive reviews tended to have written more reviews overall). Further, we checked for cross-reviewers to identify reviewers who reviewed multiple practices within the dataset.

### Statistical analysis

Descriptive analyses were conducted separately for the Practice Dataset and the Review Dataset across the four selected regions. Between-region differences were evaluated with Chi² tests for the presence of website information, listed opening hours, and special consultation hours. Practices were flagged as providing special consultation hours if they either offered opening hours (i) on Wednesday/ Friday afternoon after 2pm, (ii) on Saturday/ Sunday or (iii) on any day after 7pm. compared practices with and without missing reviews across both runs: average rating, total ratings on listing, written-review counts, and availability of website/opening hours/special hours (Mann-Whitney U for continuous, Chi² for categorical). We fit univariate proportional-odds ordinal logistic models, one model per term, with the star rating as the ordered outcome. Tokens containing only digits were excluded. For each term we report odds ratios (OR) with 95% confidence intervals (CI) and Benjamini–Hochberg [21] false discovery rate (FDR)-adjusted *q* values (α = 0.05). Analyses were run for *All Regions* and separately by region. We also ran ordinary least square (OLS) models at the practice level to examine associations of listed opening hours, special opening hours, and website with average rating. Comparative descriptive analyses were conducted to explore differences between urban vs. rural regions and Eastern vs. Western regions in Germany. To compare reviewer activity and their influence on rating behavior we used Mann-Whitney U as a robust test. All data analyses were performed using Python (v3.11) [22], utilizing *pandas* for data manipulation, *nltk* and *spaCy* for text processing, and *statsmodels* and *mord* for statistical modeling. We considered a p-value of < 0.05 statistically significant.

## Results

### Practice characteristics

A total of 1854 general practices were included in the dataset (Table 1). On average, each practice received 24.5 reviews. Opening hours were provided by roughly 1 in 2 practices (49.3%, *n* = 914), while 44.7% of practices listed a website (*n* = 829).

**Table 1:** Practice characteristics

Metric	All Regions	Lübeck	Dresden	Main-Tauber District	Mecklenburg Lake Plateau	*p*-value
total number of practices	1854	406	865	75	511	
average number of reviews per practice	24.5	28.9	24.3	18.5	22.6	
practices with opening hours listed, n (%)	914 (49.3)	197 (48.5)	431 (49.8)	55 (73.3)	231 (45.2)	< 0.001*
practices with special opening hours	207 (11.2)	31 (7.6)	114 (13.2)	13 (17.3)	49 (9.6)	0.005*
practices with website listed, n (%)	829 (44.7)	219 (53.9)	436 (50.4)	37 (49.3)	137 (26.8)	< 0.001*
first review	26-08-2011	26-08-2011	31-01-2012	11-04-2014	13-12-2013	
last review	29-03-2025	14-02-2025	29-03-2025	27-03-2025	29-03-2025	

Significant features were highlighted using asterisks

Practice characteristics showed distinct variation between regions. In the Main-Tauber District, a higher proportion of practices (73.3%) provided opening hours and special opening hours compared to the overall average of 49.3% and 11.2%. Across all regions, approximately half of the practices listed a website; the exception was the Mecklenburg Lake Plateau, where only 26.8% of practices included a website link. In a Lübeck sensitivity analysis, using the query “Hausärztin” increased distinct listings by 8.5% (union = 330), without materially changing proportions of opening hours, websites, or special hours.

### Ratings

**Fig. 1:**
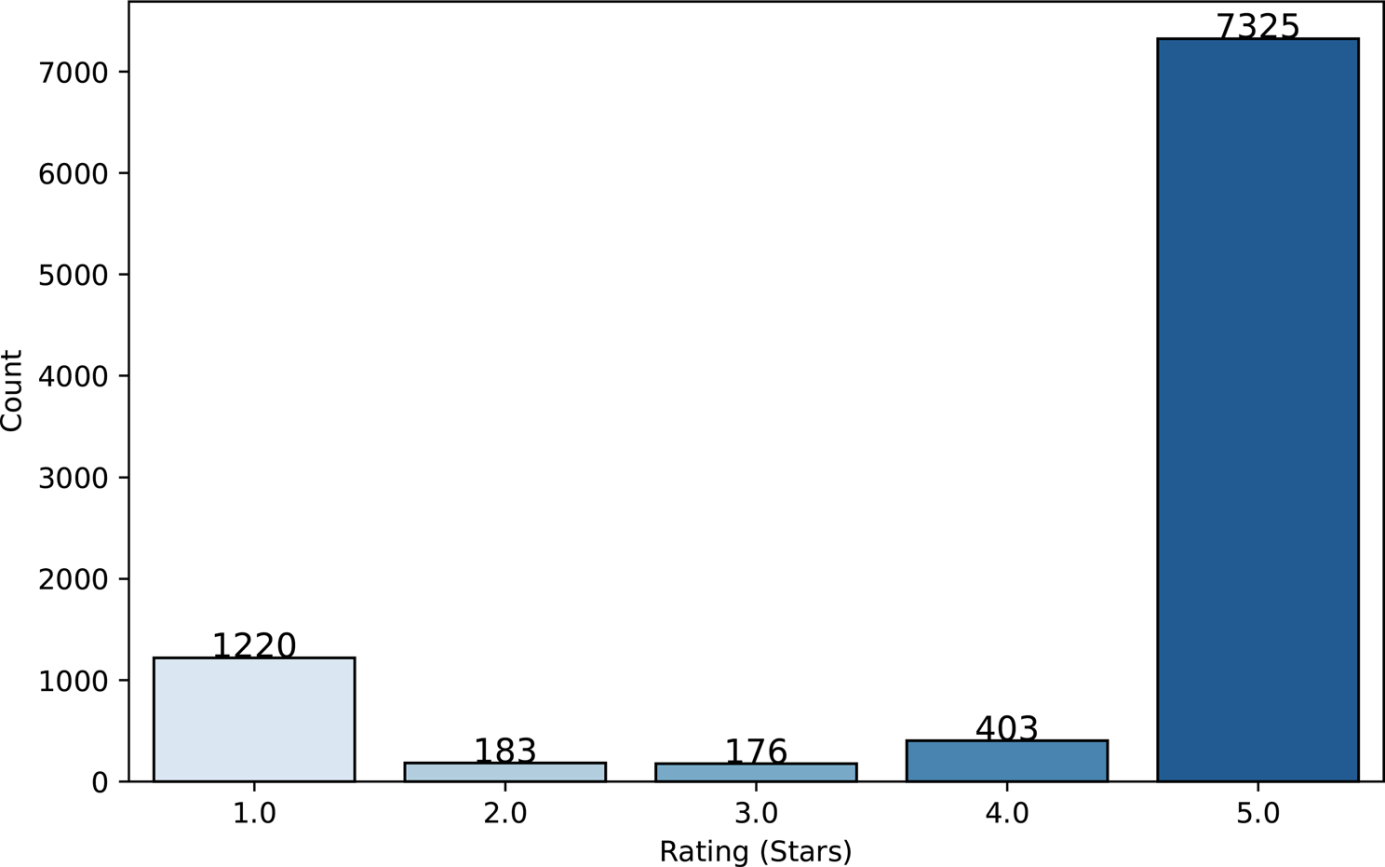
Distribution of ratings (all regions)

**Fig. 2:**
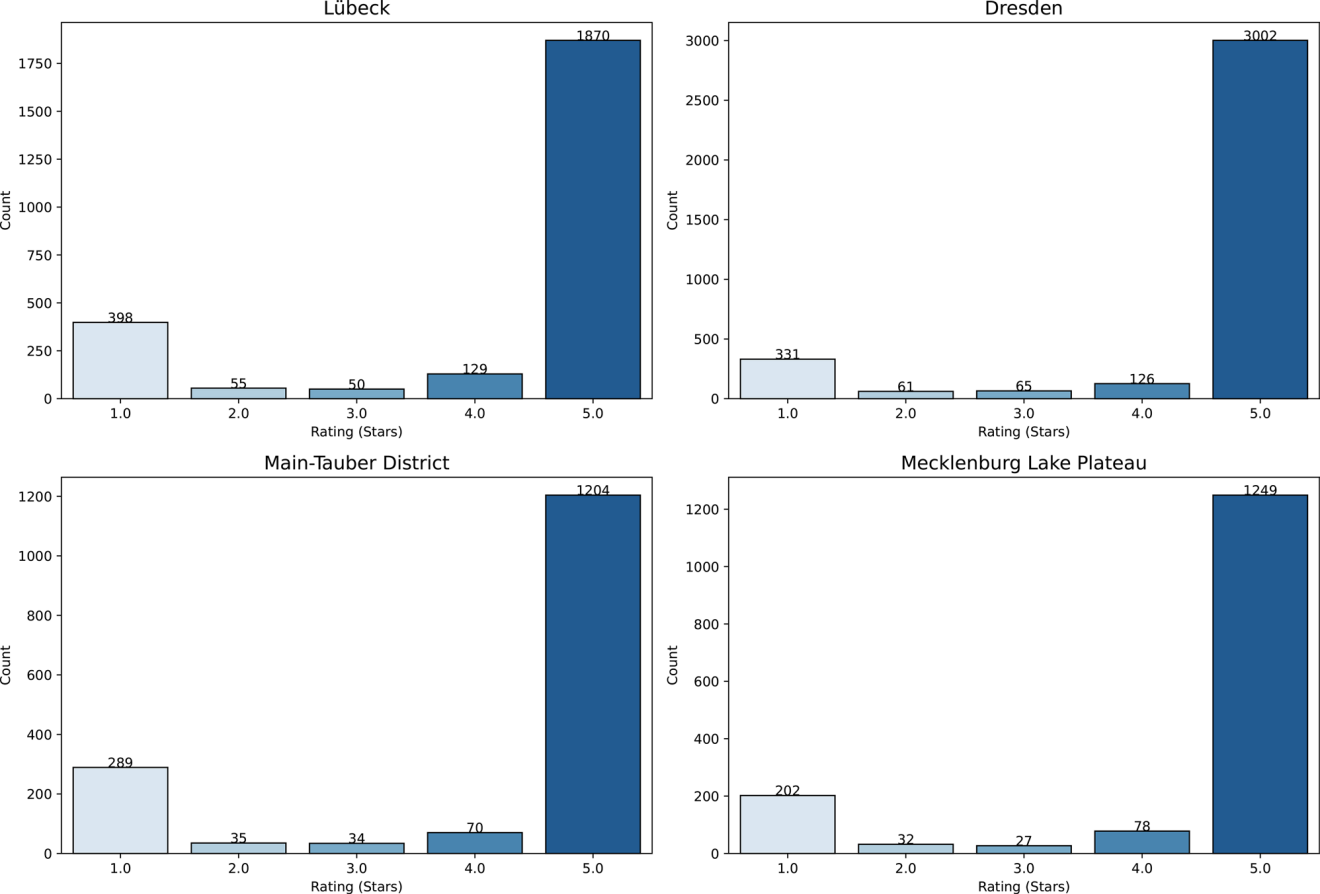
Distribution of ratings across all regions

A total of 9307 individual ratings were analyzed. The distribution of ratings was left-skewed, with 5-star ratings accounting for the majority (78.7%, *n* = 7325), followed by 1-star ratings (13.1%, *n* = 1220). Ratings with 2, 3, and 4 stars were less frequent, representing 2.0% (*n* = 183), 1.9% (*n* = 176), and 4.3% (*n* = 403), respectively (Fig. 1). The distribution of ratings was similar across all four regions (Fig. 2).

Regression models revealed regionally varying associations between practice features and ratings (Table 2). In Lübeck, the presence of opening hours was significantly associated with lower ratings (β = − 0.31, *p* = 0.004), whereas in Dresden, website presence was positively associated with rating (β = 0.17, *p* = 0.042). In Main-Tauber District, opening hours showed a strong positive association with rating (β = 0.89, *p* = 0.001). No significant associations were found in the Mecklenburg Lake Plateau. In the overall model across all regions, none of the features showed a statistically significant association with rating, although website presence showed a borderline effect (β = 0.09, *p* = 0.057).

**Table 2:** Ordinary least square regression of average practice rating on features, by region and overall

Feature	Lübeck	Dresden	Main-Tauber District	Mecklenburg Lake Plateau	All Regions
**Intercept**	4.602	4.023	3.503	4.096	4.167
Website (coef / p)	–0.047 / 0.661	+ 0.176 / 0.042*	–0.124 / 0.459	+ 0.113 / 0.237	+ 0.097 / 0.057
Opening Hours (coef / p)	–0.312 / 0.004*	+ 0.110 / 0.253	+ 0.886 / 0.001*	+ 0.157 / 0.144	+ 0.043 / 0.468
Special opening hours (coef / p)	–0.182 / 0.249	–0.068 / 0.489	+ 0.304 / 0.136	–0.013 / 0.924	–0.042 / 0.534

Significant features were highlighted using asterisks

Rerunning the query on 23 October 2025 and harmonizing practice identifiers across runs yielded *n* = 1276 practice pairs. At the review level, we could assign a harmonized practices key which resulted in *n* = 8013 Run-1 reviews and *n* = 9276 Run-2 reviews. Restricting to practices present in both runs resulted in *n* = 190 practices (Lübeck *n* = 63; Dresden *n* = 60; Main-Tauber District *n* = 30; Mecklenburg Lake Plateau *n* = 37). Among these overlapping practices, 52 (27.4%) had at least one Run-1 review missing in Run-2. By region, the share of practices with any missing review was: Dresden 38.3% (23/60), Lübeck 28.6% (18/63), Main-Tauber District 20% (6/30), Mecklenburg Lake Plateau 13.5% (5/37). Across all regions, Run-1 contained 9307 reviews and Run-2 contained 11,115. While of the Run-1 reviews, *n* = 6570 (70.6%) were also found in Run-2, *n* = 2765 (29.7%), however, were missing in Run-2. The remaining *n* = 4545 were new in Run-2.

Practices with missing reviews in Run-2 had statistically lower star rating (4.25 vs. 4.44), a higher number of ratings (48.82 vs. 27.12) and a higher number of written reviews (46.79 vs. 26.74) (Table 3). The overall star distribution was similar between missing and retained reviews (5-stars: 80.2% vs. 79.3%; 1-star: 13.0% vs. 12.3%).

**Table 3:** Practice characteristics (Run-1) by whether any Run-1 review is missing in Run-2

Characteristic	No missing reviews (*n*)	Mean (SD)	Any missing reviews (*n*)	Mean (SD)	*p*-value
Average star rating	136	4.44 (0.61)	51	4.25 (0.46)	0.001^1^
Number of ratings	136	27.12 (20.79)	51	48.82 (27.75)	< 0.001^1^
Number of written reviews	138	26.74 (20.88)	52	46.79 (25.08)	< 0.001^1^

^1^Mann–Whitney U tests (two-sided). Counts differ by row due to availability of each metric. “Number of written reviews” refers to text reviews we scraped (not star-only ratings). “Missing” means a review present in Run-1 that was not found in Run-2 for the same harmonized practice. 1276 matched practice listings across runs; 190 had ≥ 1 mapped text review in both runs and constitute the analytic panel for review overlap

### Natural language processing analysis

Frequency-based n-grams (Table 4) revealed distinct lexical patterns between positive and negative reviews. In the unigrams, frequently used positive words included *gut (good)*, *Arzt (physician)*, *freundlich (friendly)*, and *praxis (practice)*. Negative unigrams included a*rzt (physician)*, *praxis (practice)*, *gehen (go)*, and *unfreundlich (unfriendly)*. Common bigrams in 5-star reviews were *zeit nehmen (taking time)*, *aufheben gut (feel in good hands)*, and *fühlen gut (feel good)*, while 1-star reviews often contained *ernst nehmen (take seriously)*, *arzt gehen (physician go)*, and *doktor Herr (mister physician)*. Among trigrams, positively connoted phrases such as *aufheben fühlen gut (feel in good hands)* and *patient zeit nehmen (patient taking time)* were observed, whereas negative trigrams included *ernst fühlen nehmen (feel taken seriously)*, *praxis neu suchen (practice new search)*, and *gar gehen unfreundlich (go unfriendly)* (Table 4).

**Table 4:** Most frequent N-grams for positive and negative reviews

	1-Gram pos.	1-Gram neg.	2-Gram pos.	2-Gram neg.	3-Gram pos.	3-Gram neg.
1	good	physician	take time	take seriously	feel in good hands (*aufheben fühlen gut*)	feel taken seriously
2	physician	practice	feel in good hands (*aufheben gut*)	take time	patient take time	feel in good hands (*aufheben fühlen gut*)
3	friendly	go	feel good	physician go	physician take time	practice new search
4	practice	patient	physician good	physician never	take time always	practice never enter
5	take	unfriendly	friendly competent	mister physician	ear always open	even go unfriendly
6	always	say	year since	star give	practice feel in good hands	telefon direct cheeky (*telefon direkt rotzfrech*)
7	time	come	patient time	practice go	practice feel good	physician go should
8	competent	should	friendly always	practice search	since many years	friendly competent accommodating
9	feel	time	practice great	year since	always feel in good hands	physician take seriously
10	nice	more	take seriously	feel in good hands (*aufheben gut*)	since year patient	patient already many

In model-based analyses (Table 5), univariate proportional-odds regressions identified terms with strong positive or negative associations with higher ratings. Terms most strongly associated with higher ratings included *ganz team (whole team)*, *aufmerksam (attentive)*,* best (best)*,* and kompetent team (competent team).* Terms most strongly linked to lower ratings included *spiel (play)*,* unmenschlich (inhuman)*, and *unverschämt (outrageous)* (Table 5).

In total, *n* = 1762 *terms* (unigrams, bigrams, trigrams) met the inclusion threshold and were tested as independent variables in univariate ordinal logistic regressions. After correcting for multiple testing *n* = 640 remained statistically significant. The complete results for all significant terms, including ORs, 95% CIs, and FDR-adjusted q-values, are provided in Appendix A and B.

**Table 5:** Univariate proportional-odds ordinal logistic regression of n-grams (All Regions): top 10 positive and negative associations after multiple testing correction

	Term (positive association)	OR^1^	95% CI^2^	q-value	Term (negative association)	OR^1^	95% CI^2^	q-value
1	whole team	28.6	3.98–205.66	0.003	play	0.02	0.00–0.12	< 0.001
2	attentive	27.9	3.88–200.08	0.004	inhuman	0.02	0.00–0.13	0.001
3	best	22.4	3.11–161.62	0.007	outrageous	0.02	0.00–0.15	0.001
4	competent team	16.2	2.23–117.18	0.019	cough	0.02	0.00–0.16	0.001
5	friendly accommodating	14.1	1.94–102.62	0.027	incompetent	0.02	0.00–0.09	< 0.001
6	practice team	13.5	1.85–98.05	0.030	push	0.02	0.00–0.16	0.001
7	feel in good hands	13.4	5.93–30.10	< 0.001	step in practice	0.02	0.00–0.18	0.002
8	empathetic	13.3	3.27–54.17	0.001	get caught (*geraten*)	0.02	0.00–0.18	0.002
9	friendly nurse	13.2	1.84–95.19	0.031	treating unfriendly	0.02	0.00–0.18	0.002
10	dr < name of physician>	12.1	1.66–87.90	0.039	fever	0.03	0.01–0.08	< 0.001

^1^OR = Odds Ratio; ^2^CI = Confidence Interval. Q-Values represent the False Dicovery Rate (FDR) after the Benjamini-Hochberg method. Physician names were anonymized (e.g., “dr < name of physician>”)

These lexical patterns and their predictive relevance were consistent across regions, with no terms showing strong effects in only a single region. One notable exception was the term *telefon (telephone)*, which showed a negative association with ratings specifically in the Mecklenburg Lake Plateau. Isolated use of foreign language terms was observed in some regions (Appendix B).

### Reviewer analysis

The 9307 written reviews were submitted by a total of 8939 unique users. Among these, 326 users reviewed more than one practice, with 294 reviewing exactly two practices. Only two users submitted reviews in more than one of the four study regions. The group of multi-reviewers gave predominantly high ratings, with an average of 4.1 stars.

Among all reviewers, 1195 submitted a 1-star review (*“negative reviewers”*) and 7120 submitted a 5-star review (*“positive reviewers”*). On average, positive reviewers had written 26.1 reviews with text and 16.6 without text across *Google Maps*. Negative reviewers had written 14.3 reviews with text and 7.5 without text. Statistical comparisons indicated significant differences in reviewer activity between the two groups for both text-based and non-text-based reviews (*p* < 0.001) (Table 6).

**Table 6:** Positive and negative reviewers (all regions)

Metric	All Reviewers	Negative Reviewers	Positive Reviewers	All Regions – *p*-value (Neg vs. Pos)
reviews, n	9,307	1,195	7,120	-
star rating, average	4.34	1	5	-
average reviews (with text), n	25.37	14.26	26.06	< 0.001
average ratings (with/without text), n	15.99	7.54	16.57	< 0.001

## Discussion

### Main findings

This study analyzed 9307 Google reviews across 1854 general practices in four selected German regions. The vast majority of reviews were positive and the distribution was similar across all regions. Negative reviews were more often submitted by reviewers with fewer overall reviews.

NLP analysis revealed consistent lexical patterns: positive reviews frequently included terms such as *freundlich (friendly)*, *kompetent (competent)*, and *zeit nehmen (taking time)*, while negative reviews commonly referenced *unfreundlich (unfriendly)* and *unhöflich (impolite)*. While regional variation in language and rating behavior was minimal the practice features *opening hours* and *presence of a website* showed different influence on rating depending on the location. Multi-reviewers were rare and generally provided more positive ratings. While online reviews from *Google Maps* appear to reflect experiential aspects of quality (e.g., friendliness, feeling taken seriously) they function, at best, as a partial, experience-focused signal, not a comprehensive measure of care quality.

### What is already known

Online reviews of physicians are increasingly used by patients to inform healthcare decisions. Previous studies have shown that reviews on PRWs are generally favorable and often highlight interpersonal qualities such as friendliness and empathy [4–11]. The distribution of ratings on Google and similar platforms has been shown to be positively skewed in various healthcare contexts [23–28].

However, most prior work has focused either on aggregated rating [29, 30] data or specific specialties like urology [31]. Little is known about how reviewer behavior, practice characteristics, and regional contexts interact. Understanding these dynamics is important, particularly in light of concerns that fear of reputational harm or legal consequences may contribute to defensive medicine among physicians [32, 33].

### Regional variation

When compared internationally, the classification of “rural” in Germany may differ from other countries. For example, what is labeled rural in Germany often lacks the geographic isolation or resource scarcity observed in genuinely remote areas [34]. This highlights the need to interpret rurality in context when comparing international findings with mixed results, e.g. regarding workload or consultation length [35, 36].

While opening hours were positively associated with ratings in the rural Main-Tauber District and could be attributed to almost one star (β = 0.89), they were negatively associated in urban Lübeck (β = -0.31). This may reflect differing expectations or appointment structures in rural versus urban settings. However, we could not see this association in the other two regions. Further, website presence showed a small but significant effect in Dresden and a borderline effect overall, suggesting that digital visibility could support more positive evaluations in certain contexts.

Interestingly, special consultation hours were not significantly associated with ratings in any region. This may indicate that while flexible or extended hours exist, they are not systematically valued in patient feedback. Further, the two-snapshot comparison showed that the pattern of missing/retained reviews did not cluster in a single region. Additionally, our finding that patient experience is consistent across rural and urban areas is in line with a study from Japan [37].

### Patient evaluation

Linguistically, the keywords associated with positive and negative reviews were remarkably stable across urban and rural areas as well as East and West Germany in our study. This may reflect a common set of patient expectations for primary care: the physician should be friendly, competent, and professional, and both the physician and their team should take the patient seriously and make them feel well cared for. These aspects are reflected in instruments like the European Project on Patient Evaluation of General Practice Care (EUROPEP) questionnaire which provides a validated framework for assessing patient experience in primary care. The EUROPEP tool identifies two core dimensions of patient satisfaction: “clinical behaviour” (e.g., listening skills, involvement in care decisions) and “organization of care” (e.g., accessibility, helpfulness of staff) [38]. In a large-scale German study, these dimensions accounted for 60% of the variance in patient responses [39], underlining their central role in perceived care quality. In multiple studies, the same items were consistently rated highest across different populations mirroring the whole spectrum of patients in primary care: the items were “Keeping your records and data confidential” and “Listening to you” for the domain of clinical behaviour and “The helpfulness of the staff (other than doctor)” and “Getting an appointment to suit you” for the domain of organization of care [24–28]. Notably, these items closely align with the language observed in positive Google reviews in our study except “data confidentiality” which did not seem to be associated with positive reviews. Strong predictors of high ratings, like “feel in good hands”, “take time”, and “friendly” mirror the validated constructs and findings within EUROPEP [24–28, 38, 39]. While EUROPEP elicits patient experience however, via structured items, online reviews provide unsolicited narratives at scale, highlighting similar domains (communication/organization) and offering inductive language that can inform quality improvement and messaging. The second-run reinforces this interpretation: even when individual reviews drop out between scrapes, the overall star mix of missing versus retained reviews was nearly the same, suggesting the observed lexicon and rating skew are not artifacts of a single scrape.

Importantly, EUROPEP is not only descriptive but also actionable. Used in programs such as the European Practice Assessment (EPA) [40], survey results are benchmarked and fed back to practices to guide targeted improvements. Literature shows that these kinds of structured assessments are not only descriptive but can also drive improvements across several quality domains [41].

However, as pointed out by a German review, patient satisfaction might not be an ideal indicator of quality in primary care but rather serve as a measurement of service quality: as no external quality indicators were included when instruments like EUROPEP were built, higher ratings might blur unachieved outcomes relevant to patients or the amount of unnecessarily performed examinations [42]. Furthermore there is evidence, that patient satisfaction is not necessarily linked with a plus in positive outcomes, such as mortality rates [43]. However, while patient-reported outcome measures (PROM) and the concept of value-based health care (VBHC) become increasingly important in Germany [44–46], the findings of our study could inform practice management to enhance digital visibility, phone access and appointment information and to use reviews as a complementary, patient-experience lens of their practice experience.

While individual negative reviews may have strong emotional impact on practices, most reviews were positive in our study. Moreover, negative reviewers were found to write less reviews overall. This information could help practices interpret occasional negative feedback within a broader context while standardized feedback processes are missing [23]. The rating distribution found in our data is heavily concentrated at 5 stars. These ceiling effects, saturation at the top end of the response scale, however, were also found in different studies using EUROPEP [24–28], limiting discrimination among high rated practices. This might be attributable to the fact, that patients can freely choose their GP in Germany and thus select a physician they prefer.

Overall, combining structured survey instruments with real-world feedback from online platforms may offer a more complete picture of patient experience and serve as a valuable foundation for patient-centered quality improvement in primary care. Thus, our findings should be interpreted as describing patterns of patient experience rather than objective quality. Online reviews provide complementary, experience-focused signals that can inform communication, access, and team-interaction improvements. They should not be interpreted as clinical quality metrics.

### Strengths and limitations

This study has several strengths. It draws on a large, real-world dataset of public *Google reviews* and combines quantitative, linguistic, and geographic dimensions. The use of NLP methods enabled a structured analysis of sentiment-associated terms.

Limitations include the observational and cross-sectional nature of the data, which does not allow for causal inference. While the primary term “Hausarzt” captured the vast majority of listings, the gendered variant (“Hausärztin”) added a small number of additional practices in the Lübeck scrape. While practice characteristics remained stable for this region similar search terms could have an influence in other regions.

Additionally, while we could match a large proportion of practices across two runs the analysis of possible missing reviews was performed only on a small number of mapped reviews as we excluded listings without written reviews. It cannot be ruled out that deletion or disappearance of reviews occurs and our analysis indicates that both positive and negative reviews can be missing between snapshots, not only low ratings. In addition, *Google review* users may further not be representative of the broader patient population, and fake or biased reviews cannot be excluded. While for a different platform it has been shown that paid accounts receive higher ratings [47], there is no such option for *Google Maps*. Ceiling effects, however, could have masked true differences in very positive rated practices as we observed the majority of ratings to be high. Furthermore, although NLP techniques captured lexical patterns effectively, semantic nuance (e.g., sarcasm, negation) may be underrepresented. Finally, the regional selection was purposeful but limited to four areas, and results should not be generalized without caution. Further studies should, thus, may include more regions and different platforms.

## Conclusions

Most *Google reviews* for primary care practices in Germany are positive and linguistic patterns are remarkably consistent with no regional variation. Multi-reviewers are scarce and tend to give higher ratings. Strong predictors of higher ratings, such as “feel in good hands”, “take time”, and “attentive”, were similar to validated core patient expectations from EUROPEP. Thus, online reviews, offer a publicly available and easily accessible indicator of patient experience to understand patient perspectives and guide practice development. While it cannot be entirely ruled out that the deletion or disappearance of reviews influences observed patterns, our two-snapshot analysis indicates that both positive and negative reviews can be missing and that such effects do not appear to dominate the overall distribution. Therefore, these signals are complementary to, however not a replacement for, formal quality indicators.

## Supplementary Information

Below is the link to the electronic supplementary material.

Supplementary Material 1

Supplementary Material 2

## Data Availability

Data will be made available from the corresponding author upon a reasonable request.

## Abbreviations

API: Application Programming Interface
BBSR: Bundesinstitut für Bau-, Stadt-und Raumforschung
EPA: European Practice Assessment
EUROPEP: European Project on Patient Evaluation of General Practice Care
GP: General practitioner
NLP: Natural Language Processing
PROM: Patient-reported Outcome Measure
PRW: Physician Rating Website
VBHC: Value-based Healthcare
